# Social contagion of academic behavior: Comparing social networks of close friends and admired peers

**DOI:** 10.1371/journal.pone.0265385

**Published:** 2022-03-24

**Authors:** Huiyoung Shin

**Affiliations:** Department of Psychology, Jeonbuk National University, Jeonju, Chonbuk, South Korea; University of Greenwich, UNITED KINGDOM

## Abstract

Peer relations become significant socializing agents for diverse behaviors during adolescence. This study investigated relationship selection and social influence of early adolescents’ close friends and admired peers with regard to academic behavioral engagement. A stochastic actor-based model of social network analysis was used to examine classroom social networks across 2 waves (*M*_age_ = 11.46; *N* = 542) based on peer nominations. Adolescents were asked to nominate their “close friends they hang around with and talk to the most” and peers that they “admire, respect, and want to be like” Results indicated that adolescents who were similar in academic engagement more often became friends. Also, close friends’ and admired peers’ academic engagement contributed to adolescents’ own academic engagement over time. The results suggest that both close friends and admired peers are important channels for social contagion of academic behavior and that examining social relations beyond friends are important for advancing our understanding of peer social influence during adolescence.

## Introduction

Peer relations are a significant social context where adolescents’ diverse behaviors are socialized during adolescence [1]. As adolescents spend increasing amounts of time with friends and peers, characteristics of their peer social networks become immensely influential over individual’s own behaviors and beliefs [2, 3]. Extensive research has examined how and to what extent adolescents’ peer social networks affect individual beliefs and behaviors. Evidence indicates that peer environments and networks are highly influential on a variety of behaviors, including academic performance [4, 5], aggressive behavior [6], delinquency [7], and health-risk behavior [8], and that individual’s selection and influence of peers play an important role in how adolescents’ peer relations affect their adjustment [9]. Adolescents are more likely to choose peers who are similar to themselves in terms of behaviors and beliefs as friends, and these friends then serve to influence their behavior over time [10].

Although extant research has provided evidence of selection and social influence of peers for a variety of behaviors, most prior research has considered the networks of friends, and there has been limited research examining different types of peer relations. However, adolescents have peer social interactions in diverse ways, and different peer relations could have important implications for individuals’ behavioral tendencies. For example, adolescents hang around with a group of peers, cooperate on a task with a few friends, and often build close and intimate relationships with friends. Also, adolescents may admire, respect, and want to be like certain peers [11], or they could also want to be different from certain friends. Such different peer relations can play distinct roles in individuals’ behavior development, and the features of social interaction can vary by the types of individual’s social relations. Thus, specifying and examining selection and influence of peers beyond friend networks are important to advance our understanding of different peer relations’ implications for adolescents’ adjustment.

Furthermore, while the handful of studies have examined selection and influence of friends for adolescents’ academic adjustment, most prior research has focused on academic performance or achievement [5, 12, 13] and scant attention has been paid to adolescents’ actual academic behavior, such as engaged or disruptive behavior in the classroom. However, given academic performance or achievement is one of the variables that are not easily changed or influenced by other peers [5, 12], focusing on academic behavior that could lead to adolescents’ academic achievement and that can be tackled with interventions has important theoretical and practical implications.

Thus, going beyond the emphasis on adolescents’ friend networks and academic achievement in prior research, the present study investigates the implications of adolescents’ close friends and admired peers for academic behavioral engagement, which is an observable and explicitly communicated behavior that echoes individuals’ academic values and beliefs. Recent research has proposed the need to focus on “admired peers” that adolescents value and want to emulate, which is assessed with youth’s nominations of peers whom they admire, respect, and want to be like. Researchers suggested that youth’s admiration reflects what they value and desire, and their personal beliefs towards distinct attitudes and behaviors [14], and thus examining admiration, how much youth respect and want to be like particular peers, provides important insights about adolescents’ personal standards for appraising themselves and social strivings with others [11]. We assert in this study that adolescents’ academic engagement can be influenced not just by close friends but also by admired peers. By investigating (a) the extent to which similarity in academic engagement between individuals contributes to the development of friend and admire relations (i.e., relationship selection), and (b) whether academic engagement of friends and admired peers contributes to individuals’ own academic engagement (i.e., influence), the current study will provide insights into how different type of peer relations function as important channels for social contagion of academic behavior and whether there are differences in the patterns or magnitude of relationship selection and influence by the types of youth’s peer relations.

### Academic engagement among adolescents

Adolescents’ academic engagement has received much attention in several decades of research and educational practice since it plays an important role in their adjustment and development [14]. As most opportunities in current society are associated with success in school, academic engagement and achievement during adolescence can have far-reaching implications. Thus, much research has identified contributing factors of adolescents’ academic engagement, such as parents and teachers [15, 16], and recent research has also indicated that friends and peers are an important social context where adolescents’ academic values and behaviors are socialized [1]. Using longitudinal data and social network analysis, the handful of studies have indicated that adolescents tend to select friends with similar grades and level of academic competence to themselves and then over time they become more similar to their friends’ academic competence, although findings on friend influence regarding academic achievement is inconsistent or modest at best [5, 12, 13, 17]. Despite the marginal influence friends exert on academic achievement, adolescents’ friends and peers can have pervasive influence on their academic behavior. Across the school year, hanging out with peers who like school and try hard at academic work will facilitate corresponding values and behaviors, whereas hanging out with peers who find academic work boring and give little effort will dampen academic values and encourage misbehavior. However, since scant attention has been paid to adolescents’ actual academic behavior, our understanding of peer effects on academic engagement is limited in scope. If adolescents’ academic achievement is one of those costly behavior types that are not easily changed [5, 12], focusing on academic behavior that is more easily socialized and can be tackled with interventions is needed. Based on this need, we focus on relationship selection and social influence in adolescents’ academic behavioral engagement, which refers to an active, ongoing, and energized participation in academic tasks and a manifestation of the persistence and energy generated by underlying motivated psychological state [18]. Findings will provide important information about how adolescents’ peer social networks can optimize the potential of interventions to support positive and desirable academic behaviors and values.

### Close friends and admired peers

Adolescents have peer social interactions in diverse ways: They may spend leisure time with a group of peers, build close and intimate relationships with a few friends, and have admiration and respect for certain individuals [11]. Despite the variability in the types of peer relations, most research on peer social influence has focused on friend networks. Thus, the relative or cumulative importance of various social relationships sorely need greater research attention to better understand the implications of different peer relations for adolescents’ behavior and adjustment. More insights into the different effect sizes of relationship selection and social influence among various types of peer relations are vital for understanding underlying mechanisms and relevant factors shaping effective interventions.

Adolescents’ social relations operate in multiple levels. In addition to an intimate circle of close friends, they are surrounded by peers with whom they frequently interact in the classroom and social relations outside of their circle of friends has been suggested to have significant influence on adolescents’ adjustment [19]. In the peer social context, individuals with high social status have been deemed as especially influential [20, 21] since they are highly visible and more interconnected with others. Those who are popular or admired could play a central role in establishing academic or social norms [1, 22], and adolescents appraise the appropriateness of their behavior based on high-status peers’ behavior as a yardstick, leading youth to modify their own behavior to that of influential peers [23, 24]. As such, peers with high social status could have significant influence on adolescents’ behaviors, irrespective of whether they are adolescents’ close friends or not. Indeed, there has been speculation that adolescents intentionally emulate behaviors of high-status peers to promote their own social status [21, 25]. However, more research is needed to ascertain whether indeed high-status peers have pervasive influence on adolescents’ behaviors.

With attention to admired peers, the current study aimed to identify whether only close friends matter or whether admired peers are also influential for adolescents’ academic behavioral engagement. It is possible that admired peers are less influential for adolescents than close friends since social interactions with admired peers would be less frequent compared with those with close friends. In addition, given admirable peers in general tend to display more positive academic characteristics than do close friends [11], adolescents may not feel that they can be like such peers. However, it is also possible that admired peers are equally influential as close friends. Since adolescents’ admiration reflects what they value and desire, how much they respect and want to be like particular peers could provide important insight and directions for behaviors. If adolescents perceive their admirable peers’ behaviors as valuable, they may work toward developing such behaviors. Each adolescent’s idiosyncratic perceptions of admiration would define what is desirable and appropriate in terms of academic behavior, which likely to lead youth to modify their own behavior and become more like the peers they admire over time.

## Method

### Participants and procedure

We collected a dataset as part of a longitudinal investigation of early adolescents’ social networks and development; the project was approved by the IRB of the researcher’s university. The participants were fifth- and sixth- graders from public elementary schools (*M*_age_ = 11.46; 48% male in waves 1 and 2). In these schools, students were in the same classrooms with one teacher and peers for the entire day. Prior to each wave of data collection, letters explaining the current project were given to students to take home to their parents; if parents did not want their children to participate, they were instructed to have their child return an attached form to the teacher. Informed consent was obtained from all participants (i.e., teachers and students) included in the study. Teachers and students signed an assent form indicating that they understood the conditions and wanted to participate prior to starting the survey. Surveys were administered to students in their classroom when they began (Wave 1: August) and end the semester (Wave 2: December), about five months apart. Participants were assured that the information would be kept confidential and that filling out the survey was voluntary.

### Measures

#### Social networks

Participants were asked to nominate their “close friends they hang around with and talk to the most” and “peers they admire, respect, and want to be like” in the classroom. Class lists were provided for this purpose, and students could check off as many peers as they wanted. Based on the peer nominations, social networks for close friends and admired peers were calculated for each classroom. The number of participants in each social network ranged from 23 to 31. An *n* × *n* adjacency matrix was produced for each class, where *n* was the total number of classmates, with *x*_*ij*_ = 1 when there was a social tie from individual *i* to individual *j*, and with *x*_*ij*_ = 0 when there was no social tie. There was some turn-over in the participants from wave 1 to 2, so we analyzed the social networks of the 542 participants in wave 1 and their social ties that joined or left the social networks in wave 2 (by coding the missing values as structural zeros). This permitted us to control for social ties leaving and joining the networks over time [26, 27].

#### Academic engagement

Teachers reported on students’ behavioral engagement in their classroom; behavioral engagement measured the extent to which adolescents pay attention and participate in class [28]. It is composed of three items and items include “pay attention in class”, “tries hard in academic work in class”, and “listen carefully in class”. All items were rated on a five-point scale (1 = not at all true, 3 = somewhat true, and 5 = very true). The average score of the items was computed to form the composite scale, with higher scores indicative of higher engagement. Scale was reliable in the present sample (Cronbach’s α = .90 and .87 for waves 1 and 2, respectively). The validity of the scale has been established in prior studies showing concordance between student- and teacher-reported engagement.

### Analytic strategy

Analyses were conducted with a stochastic actor-based model (SAOM) of social network analysis. It is implemented in the R-based Simulation Investigation for Social Network Analysis (RSiena) software package [29]. A key strength of longitudinal social network analysis is its ability to estimate the network structural features, relationship selection, and social influence effects simultaneously [27, 30]. The first set of analyses describes various network structural features of the social networks, and the second set of analyses describes a model of network-behavioral dynamics (i.e., relationship selection and social influence). These models provide estimated parameters based on individuals’ decisions regarding changes in directed social network ties (i.e., relationship selection) and changes in their own behavior (i.e., social influence). The network and behavior dynamic parameters, which are the focus of the current study, represent the types of changes in the social relations and individual behavior over time [29].

In the current study, we estimated the network-behavior dynamics (i.e., friend/admired peer selection and their influence effects) for academic behavioral engagement, while controlling for various network structural (e.g., reciprocity and transitivity) and covariate effects (e.g., gender). We ran preliminary models separately by grade level and different random groups of classes (e.g., *N*-1 networks, *N*/2 networks). Results were consistent across grade levels and different random groups, so we combined classrooms and analyzed the dataset using the multi-group option to obtain well-converged estimates with small standard errors [29]. Analysis in SAOM yields parameters related to network dynamics (network structural and relationship selection effects) and behavior dynamics (behavioral tendencies and social influence effects). We describe in greater detail below the key aspects of what the models specified and estimated. To aid readers unfamiliar with longitudinal social network analysis, we also included detailed conceptual descriptions and graphical representations of estimated effects in S1 Table.

#### Network structural effects

To examine the network structural features, we included outdegree, reciprocity, transitive triplets, three-cycles, indegree popularity, and outdegree activity as endogenous network effects. *Outdegree* describes the overall tendency to nominate peers as close friends or admired peers. *Reciprocity* describes the tendency to reciprocate relationships. *Transitive triplets* and *three-cycles* describe the tendency to form transitive triadic relationship (e.g., my friend’s friend is my friend). *Indegree popularity* and *outdegree activity* describe the tendency to receive many nominations from others and to nominate many others, respectively.

#### Relationship selection effects

To examine the relationship selection based on engagement, we included the effects of engagement on friend/admire nominations given (*ego effects*), received (*alter effects*), and selecting similar peers on the levels of engagement (*similar behavior*). Using gender as an example for friend networks (with girls coded as 1), a positive gender ego effect indicates girls tend to nominate other peers as friends more actively than boys. A positive gender alter effect indicates girls tend to receive more nominations as friends than boys. A positive gender similarity effect indicates adolescents tend to form friendships with peers with the same gender. The effects of engagement could be interpreted in a similar manner.

#### Social influence effects

To examine the social influence of close friends and admired peers on engagement, we included *average similarity effect*. This effect estimated whether adolescents changed their engagement to more closely resemble their friends’ (admired peers’) engagement.

#### Behavioral tendencies and covariate effects

We controlled for behavioral tendencies in engagement: general tendency (*linear shape*) and dispersion (*quadratic shape*). Additionally, we controlled for potential effects from *indegree popularity* and *outdegree activity* to consider the impacts of individual’s high popularity or activity on engagement.

#### Difference between two social networks

We estimated two separate models for each social network of close friends and admired peers. Based on the estimated models, we tested for differences between parameter estimates in the network structural features, relationship selection, and social influence between two social networks with independent-sample *t*-tests, using the following formula: *(β*_*a*_*—β*_*b*_*) / √(s*.*e*^*2*^_*a*_ *+ s*.*e*^*2*^_*b*_*)*, (with estimates *β*_*a*_ and *β*_*b*_ and standard errors *s*.*e*_*a*_ and *s*.*e*._*b*_, respectively), which under the null hypothesis of equal parameters has an approximately standard normal distribution [29].

## Results

### Descriptive statistics

We first calculated the percentage overlap between close friends and admired peers using peer nomination raw data. The majority of adolescents nominated different peers concerning close friends and admired peers; We found that 26% of admired peers were included in the close friend list at wave 1 and 26.5% of admired peers were included in the close friend list at wave 2. In general, early adolescents showed the moderate levels of academic engagement at both waves (see Table 1). About 34.7% of early adolescents increased and 39.4% of early adolescents decreased for academic engagement. We presented a summary of average changes in early adolescents’ social networks from wave 1 to 2 in Table 2, and included detailed information on the variation of these statistics across separate classroom networks in S1 Data. The density indicates that adolescents nominated around 13–16% of their classmates as close friends and 9–10% of their classmates as admired peers over the school year. The reciprocity shows that about 87–91% of the nominations were reciprocal (mutual). The transitivity indicates that adolescents tend to form triadic relations; 49–53% for close friends and 38–41% for admired peers. The centrality indicates the centralization of the social networks; The proportion of all indegrees (received nominations) relative to the total number of possible degrees (in-degree centrality), was 15% for close friend networks and 14–16% for admire networks, and the proportion of all outdegrees (nominations given) relative to the total number of possible degrees (out-degree centrality), was 19% for friend networks and 2–3% for admire networks. In order to model network and behavior dynamic in SAOM with sufficient statistical power, a sufficient fraction of peer nominations should remain stable (i.e., Jaccard index). The Jaccard indices in our social networks were 42% for close friends, and 36% for admired peers. Given that a Jaccard index of 30% or more is recommended [31], the stability of our close friends and admire networks was sufficient. The goodness of fit was sufficient, and the model convergence was good (convergence ratio < .10).

**Table 1:** Changes in early adolescents’ academic engagement.

	Wave 1 –Wave 2	
Behavioral Engagement		
Mean (*SD*)	3.46 (1.03)	3.38 (0.91)
Fraction Increased	34.7%	
Fraction Decreased	39.4%	
Fraction Stable	25.9%	

**Table 2:** Changes in early adolescents’ social networks.

	Friend networks		Admired peer networks	
	Wave 1	Wave 2	Wave 1	Wave 2
Social network indicators				
*N* of students	542	514	542	514
Average (range) class size	27 (23–31)	27 (23–31)	27 (23–31)	27 (23–31)
Average *n* of social ties	4.96	4.55	3.26	3.03
^a^Density	0.16	0.13	0.10	0.09
^b^Reciprocity	0.87	0.89	0.90	0.91
^c^Transitivity	0.49	0.53	0.38	0.41
^d^Centrality (in-degree)	0.15	0.15	0.16	0.14
Centrality (out-degree)	0.19	0.19	0.02	0.03
Social network change	Wave 1–2		Wave 1–2	
Average *n* of ties dissolved	36.0 (25.2%)		33.45 (34.7%)	
Average *n* of ties emerged	51.8 (35.6%)		28.30 (29.4%)	
Average *n* of ties maintained	55.8 (39.2%)		34.60 (35.9%)	
^e^Hamming distance (change)	157		117	
^f^Jaccard index (stability)	0.42		0.36	

^a^Density is the proportion of given ties relative to the total amount of possible ties

^b^Reciprocity is the proportion of mutual ties

^c^Transitivity is the proportion of tie configurations that could become cohesive peer groups

^d^Centrality is the proportion of all indegrees or outdegrees relative to the total number of possible degrees

^e^Hamming distance is the amount of tie changes from the beginning to the end of the time point

^f^Jaccard index is the fraction of stable ties relative to all new, lost, and stable ties. Jaccard index indicates the amount of stability and should be more than 30% to permit complex SAOM with adequate statistical power.

### Longitudinal social network analyses results

Table 3 presents the longitudinal social network analyses results for early adolescents’ behavioral engagement in social networks of close friends and admired peers. To facilitate interpreting the results, we calculated odds ratios (ORs) by taking the exponential function of the parameter estimates [29]. ORs indicate the odds of the presence of a particular tendency compared with the odds of the absence of that tendency. For relationship selection, the OR indicates the odds of having similar social ties versus the odds of not having similar social ties. For social influence, having one additional social tie who reports higher than oneself increases the odds of an increase in academic engagement compared with no change. For the social influence, we first divided the parameter estimates by the number of response categories minus one to indicate the effect of a one-unit increase on the scale. ORs were not calculated for the quadratic shape effects because they are not linear. Below, we discuss our findings on the network structural effects, relationship selection, and social influence effects, which were the main results of interest.

**Table 3:** SAOM results in the social networks of friends and admired peers.

Parameter	Friends			Admired Peers		
	*B*	*SE*	*OR*	*B*	*SE*	*OR*
Network structural effects						
Outdegree	-1.72***	0.08	0.18	-1.14***	0.14	0.32
Reciprocity	1.32***	0.05	3.74	1.19***	0.06	3.29
Transitive triplets	0.54***	0.02	1.72	0.74***	0.03	2.10
Three-cycles	-0.45***	0.03	0.64	-0.26***	0.05	0.77
Indegree popularity	-0.13***	0.01	0.88	-0.03***	0.01	0.97
Outdegree activity	-0.07***	0.01	0.93	-0.42***	0.03	0.66
Network selection effects						
^a^Gender alter	-0.06	0.06	0.94	0.02	0.08	1.02
^a^Gender ego	0.06	0.06	1.06	0.09	0.09	1.09
Same gender selection	0.92***	0.05	2.51	1.26***	0.08	3.53
Engagement alter	0.01	0.03	1.01	-0.01	0.03	0.99
Engagement ego	0.01	0.03	1.01	-0.08	0.05	0.92
Similar engagement selection	0.26*	0.13	1.30	0.15	0.15	1.16
Behavior dynamic effects						
Linear shape effect	0.01	0.12	1.01	-0.34	0.27	0.71
Quadratic shape effect	-0.25***	0.05		-0.11*	0.05	
Influence	3.26***	0.61	26.05	2.32***	0.55	10.18
Indegree popularity	0.01	0.04	1.01	0.10*	0.05	1.11
Outdegree activity	0.01	0.03	1.01	0.06	0.13	1.06

^a^Gender (0 = male, 1 = female)

* *p* < .05

** *p* < .01

*** *p* < .001.

### Network structural features

**Fig 1:**
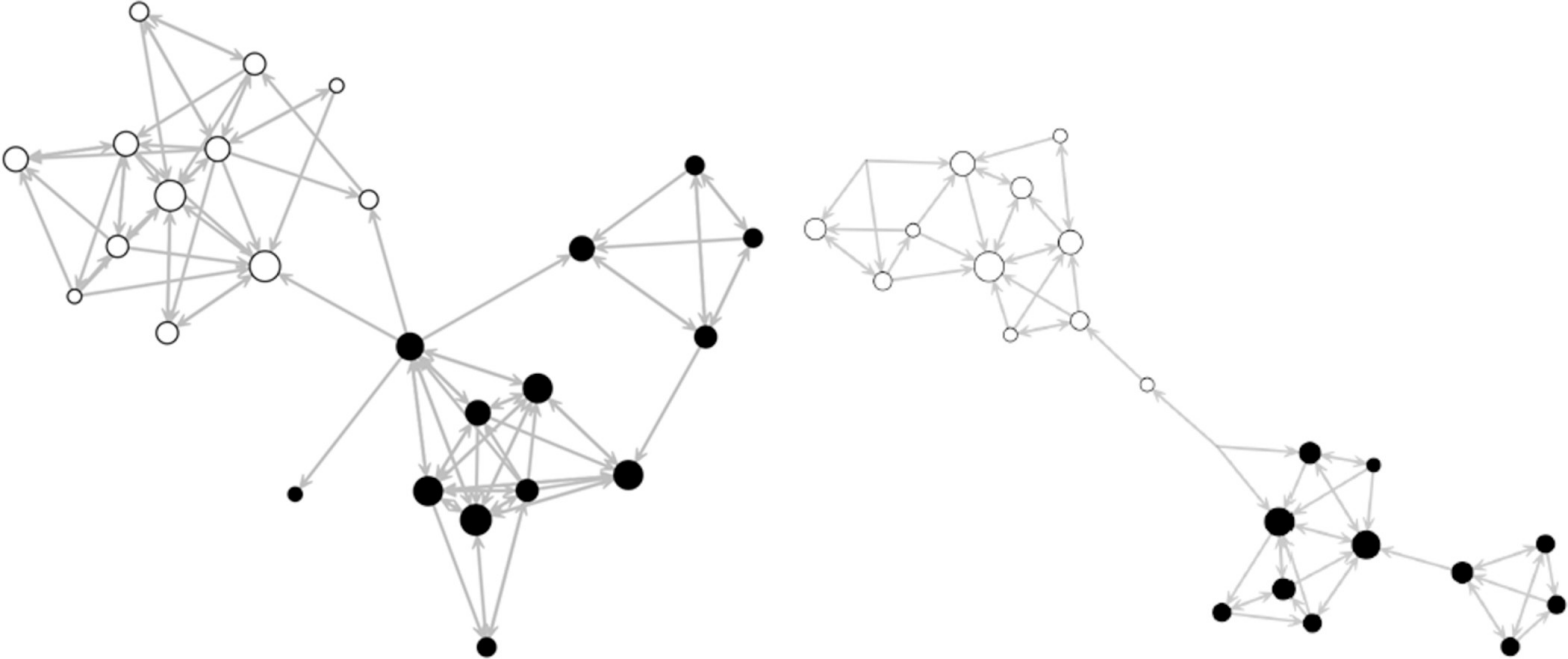
Social networks of close friends and admired peers. Social ties (arrows) are based on directed peer nominations between individuals (nods). The black nods are female, and white nodes are male. Size of the nods reflects indegrees (i.e., received nominations) of individuals.

The outdegree effect was negative and the reciprocity effect was positive for both social networks, indicating that early adolescents do not randomly nominate close friends and admired peers and tend to favor mutual social relationships. Also, positive transitivity triplets and negative 3-cycles effects were found, indicating that adolescents tend to form triadic relations (i.e., relationships with the friends of their friends) as well as cohesive group structures. The indegree and outdegree popularity effects were negative for both networks, indicating that adolescents’ social networks of friends and admired peers are not strongly centralized. Taken together, the network structural effects show that adolescents have tendencies to reciprocate relationships, keep their social networks closed and form peer group structures in their friend and admire networks. These network structural features are reflected in Fig 1, which demonstrates the sociograms of the close friends and admired peers of one classroom investigated in the current study.

### Relationship selection and social influence

Social relationships of close friends and admired peers were more likely to be formed with the same gender than different, as indicated by the positive same gender selection effect (OR = 2.51 and 3.53, *p* < .001). Regarding relationship selection, adolescents were more likely to initiate friendships with peers who had similar levels of academic engagement (OR = 1.30, *p* < .05). However, similar engagement selection effect was not significant for the admire networks, indicating that similarity in academic engagement did not affect formation of the admire relations. Regarding social influence, the positive influence effects were reflected in how adolescents’ academic engagement became more similar to those of their close friends (OR = 26.05, *p* < .001) and their admired peers (OR = 10.18, *p* < .001) over time. The difference in the magnitude of the social influence effect between the close friend and admire networks was not significant (*t* [38] = 1.14, *p* = .30). The positive indegree popularity effect for the admire networks was reflected in that adolescents who received many admire nominations showed higher academic engagement over time (OR = 1.11, *p* < .05).

To further understand the direction of relationship selection and social influence effects, we constructed ego-alter selection and influence tables for academic engagement (see Tables 4 and 5). Numbers in the Table 4 reflect the strength of friendship selection for adolescents based on their levels of academic engagement (columns dependent on rows). The values in the diagonal indicate the likelihood of friendship selection to occur when individual and friend have the same score on academic engagement. Comparing the values between rows and columns indicates that friend selection occurs between similarly low and high scoring individuals, and these effects become stronger for the higher values of academic engagement. Similarly, numbers in the Table 5 reflect the strength of social influence for adolescents to change their academic engagement based on friends’ and admired peers’ average levels of academic engagement. The values in the diagonal indicate the likelihood of social influence to occur when individual and close friends (or admired peers) have the same score on academic engagement. Comparing the values between rows and columns indicates that social influence occurs between individuals with similar scores, and these effects were the strongest for the lowest values of academic engagement.

**Table 4:** Ego-alter selection: Selection of friends (alters) on adolescents’ (egos’) engagement.

	Friends’ engagement				
Individual’s engagement	1	2	3	4	5
1	0.03	-0.02	-0.08	-0.13	-0.18
2	-0.02	0.05	-0.01	-0.06	-0.11
3	-0.07	-0.01	0.07	0.02	-0.03
4	-0.13	-0.06	0.02	0.09	0.04
5	-0.19	-0.11	-0.04	0.04	0.11

Numbers in the table reflect the strength of friendship selection for adolescents based on their levels of academic engagement (columns dependent on rows). The values in the diagonal indicate the likelihood of friendship selection to occur when individual and friend have the same score on academic engagement. The values in the cells in these tables can be transformed to odds by taking the exponential function (exp.*(β*k)).

**Table 5:** Ego-alter influence: Influence of friends and admired peers (alters) on adolescents’ (egos’) engagement.

	Individual’s engagement				
Average engagement of friends	1	2	3	4	5
1	1.46	0.40	-0.66	-1.73	-2.80
2	0.66	1.22	0.16	-0.90	-1.97
3	-0.15	0.42	0.98	-0.08	-1.15
4	-0.95	-0.39	0.18	0.74	-0.32
5	-1.76	-1.19	-0.62	-0.06	0.50
	Individual’s engagement				
Average engagement of admired	1	2	3	4	5
1	1.68	0.99	0.30	-0.39	-1.08
2	0.76	1.23	0.54	-0.15	-0.84
3	-0.16	0.31	0.78	0.09	-0.60
4	-1.08	-0.61	-0.14	0.33	-0.36
5	-1.99	-1.52	-1.06	-0.59	0.12

Numbers in the table reflect the strength of social influence for adolescents to change their academic engagement based on friends’ and admired peers’ average levels of academic engagement (columns dependent on rows). The values in the diagonal indicate the likelihood of social influence to occur when individual and friends (or admired peers) have the same score on academic engagement. The values in the cells in these tables can be transformed to odds by taking the exponential function (exp*(β*k)).

## Discussion

Moving beyond the focus on adolescents’ friend networks and academic achievement in prior research, the present study investigated the implications of early adolescents’ close friends and admired peers for academic behavioral engagement. We investigated (a) the extent to which similarity in academic engagement between individuals contributes to the development of close friend and admire relations, and (b) whether academic engagement of friends and admired peers contributes to individuals’ own academic engagement. The results indicated that early adolescents become close friends with peers who are similar to themselves in the levels of academic engagement, whereas they do not necessarily admire peers because they have similar levels of academic engagement. However, evidence that early adolescents become more similar to their connected social relations over time was found for both close friends and admired peers, and the magnitude of social influence did not differ between two social networks. The current results provide insights into how different types of peer relations function as important channels for social contagion of academic engagement and underscore that examining social relations beyond friends are important for advancing our understanding of peer social influence.

### Network structural features of close friends and admired peers

The social networks of early adolescents’ close friends and admired peers did not differ in their network structural features. Both networks were characterized by high density and reciprocity, and cohesive peer group clusters, which could be efficient in social contagion of academic engagement [32, 33]. In both social networks, early adolescents were selective in whom they formed relationships with (i.e., nominated as close friends and admired peers) and preferred reciprocal relationships with peers of the same gender. Also, both social networks were characterized by cohesive peer group structures with low centralization (i.e., nominations were not directed to a few individuals).

Empirical evidence has indicated that the strong social ties, such as close friends, that are formed within dense and cohesive social clusters are powerful in social contagion of behaviors [34]. Given that adolescents interact most regularly and intensely with their close friends, their friends would become strong social ties that could have significant social influence. It should be noted that early adolescents also formed dense social networks and peer group clusters in the admire networks. Such findings indicate that although the nominations of admired peers may be driven by adolescents’ personal beliefs and standards, their admiration may additionally depend on the network features of social interaction. For example, mutuality and peer group clusters in admire networks could occur when youth positively evaluate each other and share admiration within their groups (e.g., admire the third person through their admired peer). Collectively, the network structural features of close friends and admired peers showed that both social networks were reflected in characteristics that are efficient in social contagion of behavior [32, 33].

### Selection of close friends and admired peers

Regarding relationship selection, early adolescents tended to initiate close friendships with peers who were similar to themselves in the levels of academic engagement, but they did not necessarily admire peers because they had similar levels of academic engagement. Early adolescents wanted to be close friends with peers who were similar in the extent to which they were focused on academic work and pay attention in class. For academically engaged youth, it makes sense that they seek out friends who are also focused on academic work because friendships with similar peers could provide a safer space for them. Similar attitudes and behaviors make communication more predictable and enable connecting with less effort and with more shared feelings of belonging and understanding [35, 36].

On the contrary, similarity in the levels of academic engagement did not play a role in forming admire relationships. Adolescents would not necessarily admire peers because they have similar academic tendencies. Rather, they in general admire peers who possess some features that make them admirable. Evidence suggested that youth’s admiration is defined by characteristics that can be appreciated at a distance but have the pervasive importance within their social context, and those who display higher levels of societally sanctioned behavior such as academic engagement elicit admiration among adolescents [11, 37, 38]. Irrespective of whether youth are from economically disadvantaged environments or not, many adolescents have been shown to place high value on and express admiration for academic effort and achievement [37, 39, 40]. Insignificant similar engagement selection effect in admiration networks indicates that the reciprocal processes for friendship and admiration formation may operate differently. Similar attitudes and behaviors could be critical for friendship formation since joint activities and sharing one’s time would be crucial for close friends [41]. However, adolescents’ perceptions of admirable peers seem to be driven by a tendency to appreciate peers’ characteristics as valuable, such that adolescents admire peers who possess such positive characteristics.

### Social influence of close friends and admired peers

The results showed that both close friends and admired peers have salient social influence on early adolescents’ academic engagement, and the magnitude of social influence did not differ between two social networks. Early adolescents’ academic engagement become increasingly similar to their close friends’ academic engagement and they socialized their admired peers’ academic engagement over time. Early adolescents spend increasing amount of time bonding with their close friends sharing academic and social activities. Their intimate relationships and committing to mutually valued and shared interactions would foster socialization of academic engagement [42]. Also, adolescents’ idiosyncratic perceptions of admiration would define what is desirable and valuable in terms of academic engagement and lead adolescents to modify their own academic behavior and become more similar to the peers they perceive as admirable.

As suggested by social learning theory [23], early adolescents would use the academic behavior of their close friends and admired peers as a guideline for their own academic behavior, both in terms of ensuring its compatibility and appropriateness. And early adolescents’ circle of close friends as well as admired peers who may be outside of their friend circle, could exert significant social influence on their academic lives such that they adopt each other’s academic behavior over time. It was concerning to find that the strength of social influence was magnified when adolescents’ academic engagement was low. This is in line with prior research that suggests unhealthy attributes, such as antisocial and deviant behaviors, are most vulnerable to social contagion [43]. It indicates that early adolescents who are not focused on academic work and are distractive could experience the negative cycle through relationship selection and social influence, leading to lower levels of academic engagement. Given the positive aspects of admired peers for adolescents’ academic adjustment (e.g., admired youth showed higher academic engagement over time), researchers and educators may need to attend to the admire networks to facilitate supporting peer social dynamics in the classroom. It would be particularly important to integrate admirable peers in the informal peer interactions and motivate academically less-engaged adolescents to create social connections with highly achieving and engaged adolescents.

### Theoretical and practical implications

Findings of the current research provide evidence that academic behavior such as academic behavioral engagement can be influenced by multiple peer social relations, and that social contagion of academic engagement could be equally salient in different networks of close friends and admired peers. When adolescents have social interactions with close friends who are academically engaged and focused on academic work, their own academic engagement become more similar to that of their friends. And, admiring peers who are highly engaged in academic work increases adolescents’ own academic engagement over time. This finding is consistent with prior research examining peer relations [10] and social influence in a variety of behaviors [5, 8, 44], but the present study additionally underscores the fact that admired peers as well as close friends serve as effective channels for social contagion of academic engagement. Given adolescents’ academic achievement is one of those costly behavior types that are not easily changed or socialized [5, 12], the current findings are noteworthy.

Current study provides insights into the relative and cumulative importance of various social relations and different effect sizes of relationship selection and social influence among distinctive social relations, which is vital for understanding underlying mechanisms and relevant factors shaping effective interventions. Significant roles of admired peers can help us to better understand the social influence of high-status peers’ behavior among their classmates. Adolescents’ closeness and frequent interactions with admired peers may determine the extent to which the intervention is effective for adolescents. For example, youth could benefit more from the academic engagement intervention when they are closely affiliated with admired peers who are highly engaged and achieving. Similarly, adolescents may benefit more when they have multiple positive and less negative social relations with close friends and admired peers. Results with the current longitudinal social network analysis provide information about how interventions should be delivered to stimulate the strengthening of positive peer relationships. Specifically, admired peers could be most helpful when intervention is delivered by peer-led program [45] as high-status peers can be employed as role models who set the group norms and spread perceptions of appropriate academic behavior [46].

## Limitations and conclusion

There are several limitations that need to be addressed in future research. First, our social network measure did not take into account the quality or strength of peer relationships. The strength of social relationships is a combination of the amount of time shared together, mutual confiding, emotional intensity, and the reciprocal interactions that characterize the social tie [47]. However, we considered each social relation as equivalent in the present study. Future work that incorporates the strength of the social tie could be valuable in understanding the nature and extent of how academic behaviors and beliefs are socialized. Second, our peer nomination measures were limited to early adolescents’ classroom networks. Although this was a reasonable choice given that early adolescents spend most of their time in their classroom, it is likely that we missed their other important social relations that arise outside of their classroom. Third, owing to the small social networks in the class, our models could not be converged when we used meta-analysis. Thus, we combined classes and analyzed them simultaneously using the multi-group option, which is a typical procedure that is in line with various prior studies that considered small classrooms [48–50]. In future research with larger samples (e.g., grade-level networks), researchers could attempt to replicate the current study with meta-analyses to allow for variations between different classes. Lastly, we assessed adolescents’ social relationships at two time points, which may not capture much of the change that could occur during the school year. Future research with multiple assessments could be informative about the fluctuations in adolescents’ peer networks as well as possible relations to changes in their academic engagement.

Research utilizing longitudinal social network analysis has been burgeoning and has contributed to the understanding of the underlying mechanisms that induce the co-evolution of individuals’ social networks and diverse behaviors. To date, most prior research has investigated selection and social influence of peers without considering different types of social relations. With our study, aimed at examining different social networks of close friends and admired peers, we found that early adolescents’ academic behavioral engagement is socialized in different ways within their peer social networks. We empirically confirmed that similarity in academic engagement is a strong factor in selection of friends but not the admired peers, but both types of peer relations had salient roles in socialization of academic engagement. These findings underscore that both close friends and admired peers are important channels for social contagion of academic behavior and that specifying and examining social relations beyond friends are important for advancing our understanding of adolescents’ peer influence as well as contributing factors of social contagion.

## Supporting information

S1 Table Description of parameters used in the current models. (DOCX)

S1 File Statistics across classroom networks. (ZIP)

S1 Data Dataset. (XLSX)
