# Supplementary material for: Social contagion of academic behavior: Comparing social networks of close friends and admired peers
Source: PLoS One. 2022 Mar 24;17(3):e0265385. doi: 10.1371/journal.pone.0265385 (PMC8947254; doi:10.1371/journal.pone.0265385)
Supplement: S1 Table — (DOCX) [file pone.0265385.s001.docx]

**S1 Table. Description of parameters used in the current models.**

| Effects | Mathematical formula | Conceptual description | Graphical representation |
| --- | --- | --- | --- |
| **Network structural effects** |  |  |  |
| Outdegree | $s\text{(}x\text{) =}\sum_{j} x_{\text{ij}}\text{ }$ | Basic tendency to form social ties |  |
| Reciprocity | $s\text{(}x\text{) =}\sum_{j} {x_{\text{ij}}x}_{\text{ji}}$ | Tendency to form reciprocated social ties |  |
| Transitive triplets | $s\text{(}x\text{) =}\sum_{j,h} {x_{\text{ij}}x}_{\text{ih}}x_{\text{jh}}$ | Tendency to form social ties to friends of friends |  |
| 3-cycles | $s\text{(}x\text{) =}\sum_{j,h} {x_{\text{ij}}x}_{\text{hi}}x_{\text{jh}}$ | Tendency to form three-cycles, which is the simplest form of generalized exchange and is opposed to hierarchy |  |
| Indegree popularity | $s\text{(}x\text{) =}\sum_{j,h} x_{\text{ij}}x_{\text{+}\text{j}}$ | Tendency of actors to send social ties to those who are popular in the network |  |
| Outdegree activity | $s\text{(}x\text{) =}\sum_{j,h} x_{\text{ij}}x_{\text{i}\text{+}}$ | Tendency of actors who nominate many others as social contacts to nominate more social ties |  |
| **Selection effects** |  |  |  |
| Alter effect | $s\text{(}x\text{) =}\sum_{j} \upsilon_{\text{j}}x_{\text{ij}}$ | Tendency to receive social ties based on the individual’s mean level on the attribute |  |
| Ego effect | $s\text{(}x\text{) =}\text{ }\upsilon_{i}\sum_{j} x_{\text{ij}}$ | Tendency to select social ties based on the individual’s mean level on the attribute |  |
| Similar behavior | $s\text{(}x\text{) =}\sum_{j} x_{\text{ij}}\text{(sim}_{\text{ij}}^{v}-\hat{\text{sim}^{v}})$ | Tendency to select each other based on similarity between the individual and social ties in the attribute |  |
| **Influence effects** |  |  |  |
| Linear shape effect | $s_{i}^{\text{beh}}(x, v)=v_{i}$ | Tendency toward high or low values on the attribute |  |
| Quadratic shape effect | $s_{i}^{\text{beh}}(x, v)=v_{i}^{2}$ | Tendency of self-reinforcing (positive estimate) or self-correcting (negative estimate) effect |  |
| Average similarity effect | $s_{i}^{\text{beh}}(x,v)=x_{i+}^{-1}\sum_{\text{j}} x_{\text{ij}}(\text{sim}_{\text{ij}}^{v}-\hat{\text{sim}^{v}})$ | Tendency to become more similar in the attribute over time: social tie’s attribute predicts changes in the individual’s attribute |  |

SAOM provides estimates of the objective functions for the network (Eq 1) and behavioral changes (Eq 2), which are calculated as a linear combination of the effects where $\beta$_k_ are the parameters and s_ik_ ($x$) are the effects:

$f_{i}^{\text{net}}(x)=\sum_{k} \beta_{k}^{\text{net}}s_{ik}^{\text{net}}(x)$ (Eq 1)

$f_{i}^{\text{beh}}(x, v)=\sum_{k} \beta_{k}^{\text{beh}}s_{ik}^{\text{beh}}(x, v)$ (Eq 2)

The objective functions for the network consist of a set of parameters that reflecting the network structural features and the network selection effects. The objective functions for the behavior consist of a set of parameters reflecting the behavioral tendency and social influence effects. S1 Table describes in greater detail the key aspects of what the models specified and estimated; A social tie from actor i to actor j is called $x_{\text{ij}}$. The variables of $x_{\text{+j}}$ and $x_{\text{j+}}$ indicates all incoming and outgoing social ties of actor j, respectively. Variable$\upsilon_{\text{i}}$ refer to the attribute of actor i, and $\hat{\text{sim}^{v}}$ is the mean of all similarity scores. Information was derived from RSiena manual.
